# Evaluation of absorption and depletion of florfenicol in European seabass *Dicentrarchus labrax*

**DOI:** 10.1002/vms3.415

**Published:** 2020-12-23

**Authors:** Dimitra Kogiannou, Chrysanthi Nikoloudaki, Pantelis Katharios, Adriana Triga, George Rigos

**Affiliations:** ^1^ Hellenic Centre for Marine Research (HCMR), Institute of Marine Biology, Biotechnology and Aquaculture (IMBBC) Anavyssos Attika Greece; ^2^ Hellenic Centre for Marine Research (HCMR) Institute of Marine Biology, Biotechnology and Aquaculture (IMBBC), Former American Base of Gournes Heraklion, Crete Greece

**Keywords:** florfenicol, florfenicol amine, muscle plus skin, pharmacokinetics, withdrawal time

## Abstract

The pharmacokinetic properties and residue elimination of florfenicol (FLO) and its amine were investigated in European seabass *Dicentrarchus labrax* at 24°C. The trial mainly included analysis of FLO in plasma after a single dose dietary administration of 10 mg/kg and in muscle plus skin following a multiple dosing (10 mg kg^−1^ day^−1^ for 7 days) to estimate pharmacokinetics and residue depletion, respectively. The maximum plasma concentration of FLO was measured to be 1.64 μg/ml, 4 hr post administration. The elimination half‐life (*t*_1/2b_) and the area under the concentration‐time curve extrapolated to infinity (AUC_0‐∞_) were calculated to be 13.0 hr and 34.7 μg h^−1^ ml^−1^, respectively. Withdrawal times of FLO and its amine were calculated to be 46.3 degree‐days, indicating a fast removal from the edible tissues of treated European seabass. Overall, FLO can be considered as a potentially efficient antibacterial agent for farmed European seabass provided that additional efforts will be devoted towards its in vitro and clinical efficacy.

## Graphical abstract

First pharmacokinetic study of florfenicol in European seabass providing insights to enhance treatment schedule of a promising “off label” antibacterial compound. Information is also provided regarding consumer's safety.

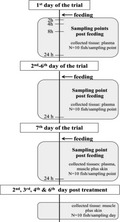

## 1 INTRODUCTION

Along with gilthead seabream *Sparus aurata*, European seabass *Dicentrarchus labrax* is an economically important species in Mediterranean marine fish farming, with production reaching 191,000 tons in 2016 (EUMOFA, 2018). In the grow‐out stage, the main farming practises of European seabass make use of cage facilities where a large number of growing animals are engaged in a 12‐month struggle for life against several different stressors. The ensuing disease outbreaks can become a serious economic hazard for caged European seabass. Proper health management practices and high standards of hygiene are the most effective preventive control methods to reduce the risks of a disease outbreak. Among the potential disease sources, bacterial pathogens are perhaps the most frequently encountered for European seabass (Colorni & Padros, 2011). Despite the preventive strategies adopted in aquaculture enterprises, bacterial outbreaks are occasionally inevitable especially when commercial vaccination is lacking and thus, in those cases the use of antibacterial compounds appears as the sole option.

In most Mediterranean countries, only a few antibacterial compounds are licensed for aquatic medical use (EC^470^/, 2009; EC^37^/, 2010; Rigos & Troisi, 2005). A narrow range of available antibacterial compounds may however trigger the spread of antibiotic resistance, thus reducing the available therapeutic options (Watts et al., 2017). The search of additional antibacterials is consequently of primary importance for aquatic medicine practiced in this region. Florfenicol (FLO), a synthetic amphenicol, could potentially be an effective solution to the limited range of established antibacterials.

Florfenicol is related to chloramphenicol and exerts broad spectrum antibacterial activity (inhibition of protein synthesis) against gram‐negative bacilli, gram‐positive cocci and other atypical bacteria (Papich, 2016). It is more active however than either chloramphenicol or thiamphenicol and can even display bactericidal action (Papich, 2016). Moreover FLO is highly lipophilic, and can provide concentrations high enough to treat intracellular pathogens and easily crosses biological barriers. Florfenicol has been entered into Annex I of Council Regulation (EC) No 2377/90 with a maximum residue level (MRL) of 1,000 μg/kg fish muscle plus skin (EMEA, 2002). The compound is registered for aquatic use in few Mediterranean countries, while it can be prescribed as a drug authorised for other than fish farmed animals by the prescribing cascade mechanism (90/676/EC), where the compound is not labelled. In such cases, a standard withdrawal period is imposed, corresponding to 500 degree‐days (dd) in fish (Directive 2004/28/EC).

Florfenicol has been clinically assessed (Gaunt et al., 2003; Soto et al., 2010) and its absorption after oral administration has been investigated in several important marine farmed fish species including Atlantic salmon *Salmo salar* (Horsberg et al., 1996; Martinsen et al., 1993), turbot *Scophthalmus maximus* (de Ocenda et al., 2017), orange‐spotted grouper *Epinephelus coioides* (Feng et al., 2018) and hybrid striped bass *Morone chrysops* × *M. saxatilis*. The absorption of FLO has been also determined in main freshwater farmed fish species such as Nile tilapia *Oreochromis niloticus*, hybrid tilapia *O. niloticus* × *O. aureus* (Feng & Jia, 2009; Kosoff et al., 2009), rainbow trout, *Oncorhynchus mykiss* (Pourmolaie et al., 2015), channel catfish *Ictalurus punctatus* (Gaunt et al., 2012, 2013) and crucian carp *Carassius auratus* (Zhao et al., 2011). However, pharmacokinetic information of FLO is totally lacking in European seabass regardless of its commercial importance in Mediterranean marine fish farming. Therefore, the aim of the present work was to provide insights into important pharmacokinetic parameters for dosing schedule, such as absorption and depletion, in European seabass following a single and a multiple oral FLO administrations at water temperatures optimum for bacterial outbreaks.

## 2 MATERIALS AND METHODS

### 2.1 Experimental fish

Apparently healthy European seabass averaging about 100 ± 12 g were obtained from a local fish farm (Selonda aquaculture S.A) and distributed in two cages (1 m^3^) located within a 50 m^3^ cement tank (85 fish/cage). Water was supplied by open flow and oxygen was provided continuously by bubbling air. The water temperature and salinity were 24 ± 1°C and 38‰, respectively. Fish were allowed to acclimate for 7 days prior to experimentation and fed a drug‐free commercial diet at 1.5% body weight (B.W.). To increase acceptance of the therapy, fish were starved for 24 hr prior to administering the medicated feed. Management of experimental animals followed the EU legislation ‘on the protection of animals used for scientific purposes’ according to the EC Council Directive 2010/63/ EU (EU, 2010).

### 2.2 Medicated feed and drug administration

Fish received a commercial feed (BioMar, Denmark) (Table 1) with oil‐coated FLO (Nuflor, Merck animal health, USA), aiming to simulate an in situ preparation of a medicated diet. One batch of 2 kg of medicated feed was prepared by mixing 2 kg of feed, FLO (1.35 g of active compound or 4.5 ml of Nuflor, 30% active) and 100 ml fish oil for several minutes. During the trial, the experimental diet was stored at 4°C and was left to obtain ambient temperature before delivery. Fish were fed the experimental diet by hand once per day for seven consecutive days at 1.5% B.W. The amount of feed offered was calculated on a daily basis, depending on the number of fish remaining in the tanks. The dose of administered FLO was estimated to be 10 mg/kg fish/day.

**TABLE 1:** Composition of the commercial diet

Proximate composition	g/100 g
Protein	41–43
Lipid	18–20
Nitrogen‐free extract	16–22
Fibre	1.9–4.9
Ash	8–10
Total phosphorus	1.2–1.4

### 2.3 Sampling

**FIGURE 1:**
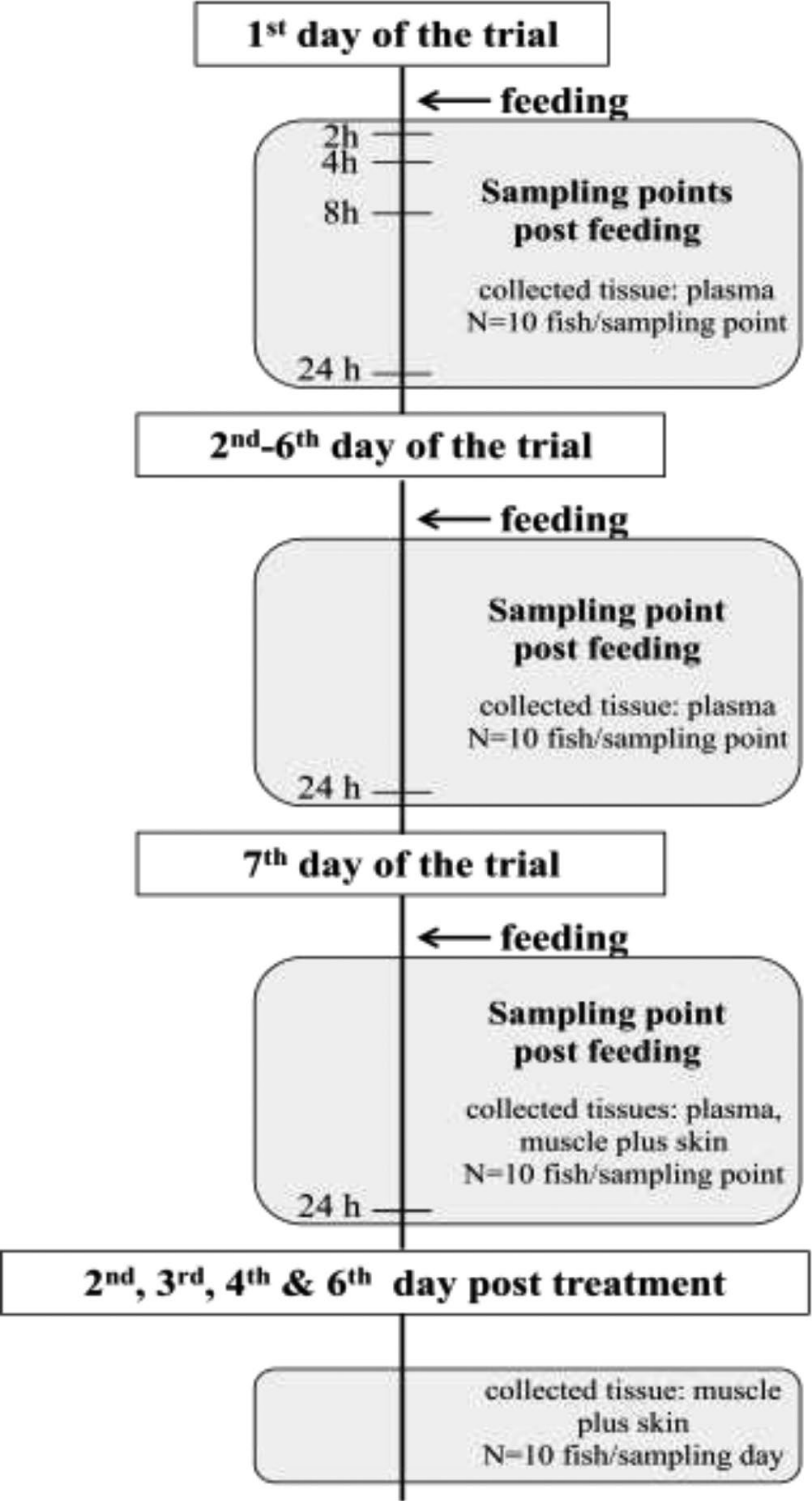
Study design (feeding strategy and samples collection) for the investigation of florfenicol (FLO) absorption and depletion profiles in European seabass held at 24°C following a single (10 mg/kg) and a multiple oral dosing (10 mg kg^−1^ day^−1^)

Sampling of fish was performed at predetermined time points during and post treatment (Figure 1) after anesthetization with clove oil (40 mg/L). Sampling of plasma during the first 24 hr was dedicated to FLO pharmacokinetics while sampling in the remaining experimental days was devoted to daily plasma measurements (during treatment) and residue depletion (post‐treatment). During the first day, blood samples were collected at 0, 2, 4, 8 and 24 hr. On the other intervention days during therapy, that is, from day 2 to day 7 of experimentation, blood was daily sampled at 24 hr post administration. At each sampling point, 10 fish were killed by a blow to the head and tissue samples were obtained. Approximately, 2 ml of blood was drawn with a needle (Microlance 23G 11/4 0.6 × 30; Becton Dickinson, Zaragoza, Spain) from the caudal vein at selected time points during treatment and transferred into heparinised test tubes. Plasma was separated from blood samples by centrifugation at 20,160 *g* for 10 min at 4°C. Moreover for the withdrawal study, muscle plus skin (approximately 5 g) were taken from the anterior dorsal region on days 1, 2, 3, 4 and 6 post treatment. All prepared tissue samples were immediately frozen and stored at −20°C until analysis.

### 2.4 Chemicals and reagents

Analytical standards of FLO and its amine were obtained from Sigma‐Aldrich (USA). High‐performance liquid chromatography (HPLC) grade acetonitrile and ethyl acetate were purchased from Fisher Scientific (USA). Other reagents of analytical grade were supplied by Fisher Scientific (USA), while heparin (5,000 IU/ml) was obtained from Merck KGaA (Germany). Stock solution of 100 μg/ml FLO and its metabolite were prepared by dissolving FLO and its amine in acetonitrile and stored at −20°C. Fresh working solutions (10 μg/ml) of each compound were prepared before dilution with acetonitrile:water (35:65 v/v). Working solutions were further diluted with acetonitrile:water (35:65 v/v) for calibration curves.

### 2.5 Sample preparation

The extraction and analysis of FLO in plasma samples was carried out according to the procedure of Xie et al. (2011). Briefly, 1 ml of plasma sample was placed in a 10 ml polypropylene centrifuge tube with 500 μl of acetonitrile:water (35:65, v/v). The mixture was vortexed for 30 s, followed by the addition of 5.5 ml of ethyl acetate to deproteinize and extract the FLO. It was then mixed for 2 min and homogenized ultrasonically for 15 min. The homogenised samples were centrifuged (8,000 *g* for 10 min) and the supernatant was transferred to a 15 ml polypropylene centrifuge tube. The extraction step was repeated twice. The combined extract was then evaporated to dryness at 40°C under a gentle stream of nitrogen. The residue was reconstituted by 1 ml of mobile phase solution. Five milliliter of hexane were added into the tube and after mixing it was subjected to centrifugation for 5 min at 2,150 *g* prior to the removal of the hexane layer. The afore‐mentioned de‐fatting step was also repeated twice. The water‐based phase was filtered using 0.22 μm nylon filter and the filtrate (200 μl) was then analysed by HPLC.

A modified method of Feng et al. (2008) was used for FLO extraction and analysis in muscle samples. Briefly, muscle plus skin samples were sheared, and subsequently 2 g of ground sample was weighed into a 50 ml centrifuge tube. Ten ml of ethyl acetate were added and the mixture was homogenized with an IKA Ultra‐Turrax T25 Disperser (IKA^®^‐Werke GmbH & Co. KG, Staufen, Germany) for 30 s at 16,000 *g*. The mixture was agitated for 20 min and then was centrifuged at 3,500 rpm/min for 10 min at 5°C. The supernatant was transferred to a 15 ml polypropylene centrifuge tube and the extraction steps were repeated twice. The combined extract was then evaporated to dryness at 45°C under nitrogen stream. Two milliliter of hexane and 1 ml of mobile phase solution were added in the residue. After gentle agitation for 5 min, the mixture was centrifuged (2,150 *g* for 5 min). The upper layer (hexane) was discarded and the de‐fatting step was repeated. The bottom layer (1 ml) was filtered (0.22 μm nylon filter) and then was subjected to HPLC analysis.

### 2.6 HPLC analysis

Chromatographic separation of parent compound and its amine was carried out in a HPLC apparatus combining a 600 Pump system Controller with a 600 Pump comprising a column heater, a 717 Plus Autosampler set at 10°C injection temperature, a 250 mm × 4.6 mm Symmetry‐C18 column packed with 5 μm particle size, a 470 fluorescence detector set at 224 nm for excitation wavelength and 290 nm for emission wavelength and an Empower Chromatography Software (all from Waters, Milford, MA, USA). The column temperature was maintained at 30°C and the injected volume was 200 μl. As a mobile phase, (A) an aqueous solution of NaH_2_PO_4_ (0.01 M) containing 0.005 M sodium dodecyl sulphate and 0.1% triethylamine (pH 4.8 adjusted with 85% phosphoric acid) and (B) acetonitrile were used (A/B, 65:35, v/v). The flow rate was 1.0 ml/min.

To establish the calibration curves for quantification of FLO concentration in plasma and muscle plus skin samples as well as FLO amine concentration in muscle plus skin samples, the FLO or/and its amine standards were spiked into blank European seabass plasma and muscle plus skin samples at final concentrations of 0.01, 0.05, 0.1, 0.25, 0.5 1, 2.5, 5, 10 μg/ml or g (analyses were performed in pentuplicate). For the determination of the drug and its major metabolite from the spiked samples, the extraction procedure and HPLC method described above were used. To evaluate the recovery rates of FLO and FLO amine and the intra‐ and inter‐day coefficients of variation (CV), three replicates of spiked samples (plasma and muscle plus skin) containing different concentrations of the substances (0.1, 1 and 10 μg/ml or μg/g) were examined for two days. For quantification, the peak area measurements were used. The recovery of the methods was calculated by comparing the determined concentration of spiked samples with those of standard solutions. The limits of detection (LOD) and the limits of quantification (LOQ) were calculated by 3.3*σ/S and 10*σ/S, respectively (σ = standard deviation of the y‐intercept of the regression line; S = slope of the calibration curve).

### 2.7 Pharmacokinetic parameters

Calculation of the pharmacokinetic parameters of FLO in European seabass plasma after a single dietary administration of FLO (10 mg/kg) was carried out by the non‐compartmental pharmacokinetic model based on the statistical moment theory, according to the method described by Gibaldi & Perrier (1982). The maximum plasma concentration (observed maximal concentration) and the time to reach maximum plasma concentration were measured directly from the mean plasma drug concentration versus time profiles. A semi‐logarithmic graph of mean plasma concentration at the elimination phase versus time was used for the elimination half‐life (*t*_1/2β_ = 0.693/β) calculation. The area under the concentration–time curve (AUC_0‐∞_) was determined using trapezoidal method and was extrapolated to infinity. Calculation of the total body clearance (*Cl_T_*/*F*) was also performed in a model independent way (Ritschel, 1986).

### 2.8 Withdrawal times (WTs)

Withdrawal times were calculated based on the guidelines of European Medicines Agency (EMA) (2018). The total concentrations of FLO and its amine in muscle plus skin were calculated at each sampling time point post treatment and subjected to a linear regression analysis versus time data from each individual using the statistical program WT1.4 (Hekman, 2004). Withdrawal period was determined at the time when the upper one‐sided 95% tolerance limit for the residue was below the MRL with 95% confidence.

### 2.9 Statistical analysis

Results are presented as means ± *SD* (standard deviation of the mean). Mean plasma concentrations of FLO of each intervention day were compared using one‐way analysis of variance (ANOVA), while levels of significance were set at *p < *0.05. The SPSS version 25.0 (International Business Machines Corporation, Armonk, NY, USA) was used for the statistical analysis.

## 3 RESULTS

### 3.1 HPLC method

A linear relationship for both FLO and FLO amine existed in the calibration curves over the range of 0.01–10 μg/ml of plasma and muscle plus skin tissues, and the coefficients of correlation were greater than 0.998 (*R* = 0.9997 and *R* = 0.9988, respectively). The retention time of FLO in plasma samples was 7.7 min and the retention times of FLO and its amine were 7.8 and 10.8 min for the muscle plus skin homogenate, respectively. The recovery rates of FLO were 94%–101% and 92%–101% for plasma and muscle plus skin samples, respectively, while the recovery rates of FLO amine in muscle plus skin tissues were 95%–98%. The intra‐ and inter‐day coefficients of variation of FLO and FLO amine in tested tissues were measured to be below 6.9%. The LOD of FLO was set to 0.02 μg/ml and 0.03 μg/g in plasma and muscle plus skin (FLO and amine), respectively, while the respective values of LOQ were 0.03 μg/ml and 0.05 μg/g for the two analysed tissues.

### 3.2 Pharmacokinetics of FLO

**FIGURE 2:**
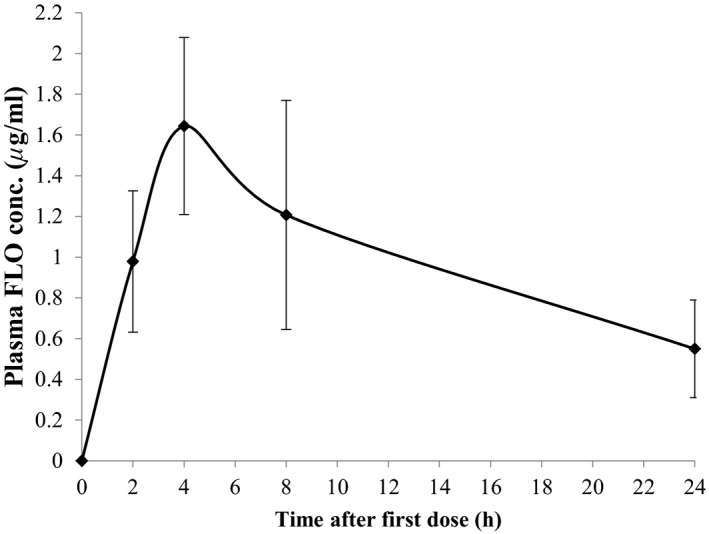
Mean plasma concentrations of florfenicol (FLO) following single (the first experimental day) oral administration of 10 mg/kg in European seabass held at 24°C. Mean ± *SD* are shown (*N* = 10)

**FIGURE 3:**
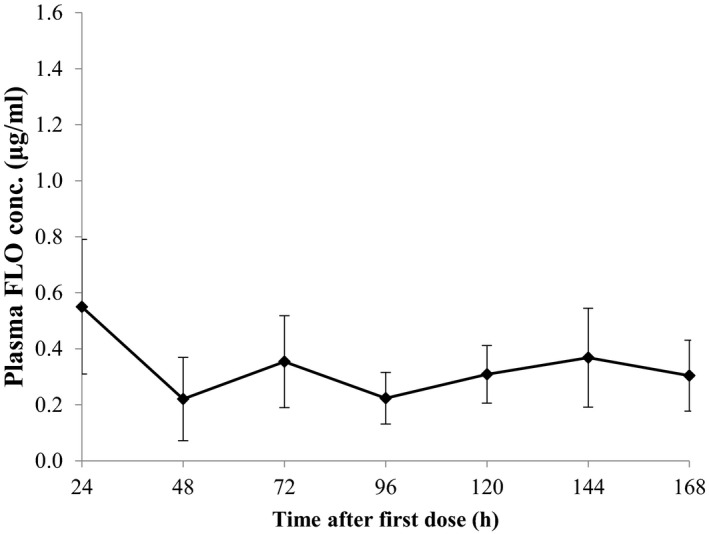
Minimum plasma concentrations of florfenicol (FLO) after multiple oral administrations at 10 mg/kg per day for seven consecutive days in European seabass held at 24°C. Mean ± *SD* are shown (*N* = 10)

During the 7‐day treatment, fish readily consumed the entire quantity of medicated diet offered with no obvious signs of lack of appetite. Mean concentrations of FLO in European seabass plasma during the first 24 hr after receiving a single dietary administration of FLO (10 mg/kg) are shown in Figure 2. The maximum plasma concentration (1.64 μg/ml) was measured directly from the mean plasma drug concentration versus time profiles (Table 2) which was detected at 4 hr post drug administration. Drug level declined at a level of 0.55 μg/ml at 24 hr post medication. The elimination half‐life (*t*_1/2b_), the *Cl_T_*/*F* and the AUC_0‐∞_ were calculated to be 13.0 hr, 0.29 L kg^−1^ h^−1^ and 34.7 μg h^−1^ ml^−1^, respectively (Table 2). Minimum plasma concentrations of FLO at the 24 hr sampling intervals after multiple oral administrations at 10 mg/kg per day for seven consecutive days are presented in Figure 3. Values ranged from 0.22 to 0.55 μg/ml; however, no statistical difference between time intervals was found.

**TABLE 2:** Pharmacokinetic parameters of florfenicol (FLO) after oral administration at 10 mg/kg in European seabass held at 24°C

Parameter	
Maximum concentration (μg/ml)	1.64
*t*_1/2b_ (h)	13.0
AUC_0‐∞_ (μg h/ml)	34.7
*Cl_T_*/*F* (L kg^−1^ h^−1^)	0.29

Abbreviations: AUC_0‐∞_, area under the drug concentration curve extrapolated to infinity; *Cl_T_*/*F*, total body clearance of the drug divided by bioavailability (*F*); *t*_1/2b_, elimination half‐life of the drug.

### 3.3 Depletion and WTs of FLO

**FIGURE 4:**
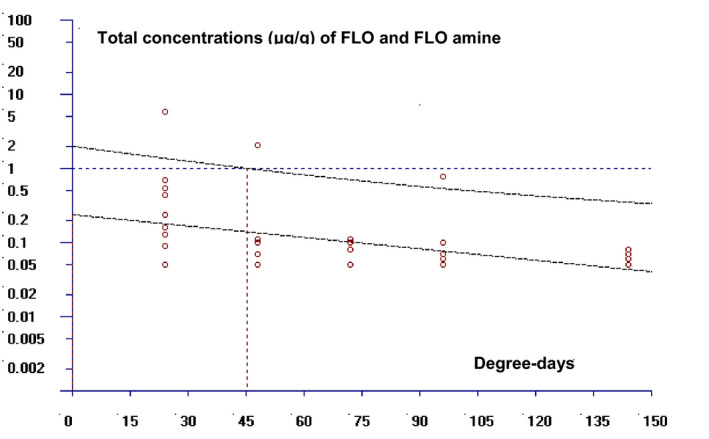
Plot of the WTs calculation of florfenicol (FLO) and its main metabolite FLO amine in European seabass muscle plus skin at the time when the one‐sided 95% upper tolerance limit is below the MRL for FLO (1,000 μg/kg) after multiple oral administration of 10 mg/kg per day for seven consecutive days (*N* = 10)

Mean and standard deviations of the parent FLO and its amine residues in muscle plus skin samples are presented in Table 3. The results indicate that the elimination of FLO and its metabolite in edible tissues were rapid as the drug concentrations declined below LOQ 144 hr post treatment. The WTs for of FLO and its metabolite in European seabass muscle plus skin tissues (Figure 4) were calculated to be 46.32 dd.

**TABLE 3:** Muscle plus skin concentrations of florfenicol (FLO) and its amine after multiple oral administrations at 10 mg kg^−1^ day^−1^ for seven consecutive days in European seabass held at 24°C. Data present mean ± *SD*

Hours after last dosing	Muscle plus skin FLO concentration (mg/kg)	Muscle plus skin FLO amine concentration (mg/kg)
24	0.80 ± 1.74	0.06 ± 0.04
48	0.25 ± 0.64	<LOQ
72	0.04 ± 0.02	<LOQ
96	0.04 ± 0.01	<LOQ
144	0.03 ± 0.01	<LOQ

Abbreviation: LOQ, limits of quantification.

## 4 DISCUSSION

This is a preliminary pharmacokinetic study of FLO in European seabass which provides valuable information for the design of efficient treatment schedules and for ensuring consumer safety in this species. The maximum plasma concentration value of FLO in European seabass was found 1.6 μg/ml which is admittedly lower compared to those calculated after dietary delivery in other farmed fish species (1.8–55 μg/ml) held however in different environmental conditions (Table 4). Apparently, as with most pharmacokinetic parameters, maximum plasma concentration values are strongly interspecific as well as experimentally and environmentally dependent (Chang et al., 2019; Feng et al., 2008; Huang et al., 2019; Rairat et al., 2019, 2020) and therefore, should be seriously considered when direct comparisons are attempted among different studies. Water temperature (Chang et al., 2019; Huang et al., 2019; Rairat et al., 2019) and salinity (Feng et al., 2008; Rairat et al., 2020) in particular have been specifically demonstrated as substantial factors affecting the kinetic profile of FLO in farmed fish. In fact, increasing water temperature would cause an increase in blood flow and in drug metabolic rate, leading to enhanced clearance of the drug (Rairat et al., 2019).

**TABLE 4:** Published florfenicol (FLO) pharmacokinetic studies after oral administration in farmed fish

Species	Salinity	Administration route	Dose (mg/kg fish)	Size (g)	Temperature (^o^C)	*C*_max_ (μg/ml)	*T*_max_ (h)	*t*_1/2b_ (h)	WT (h)	References
Tilapia	0‰/16.5‰	SO‐FA	10	115	28					Feng et al. (2008)
	0‰	SO‐FA	10	98	22	4.5	12	10.0		Feng and Jia (2009)
Nile tilapia	0‰	MO	15 × 10d	100–500	25	7.1–15.2		1.2–2.2	>288	Bowser et al. (2009)
			10 × 10d	444–474	25–30	6.5–6.7			98–146	Kosoff et al. (2009)
		SO‐FA	15	500–700	24–28–32	23.1–16.7–16.7	1.4–1.2–0.8	12.4–9.5–7.3		Rairat et al. (2019)
		SO‐FA	15	500–700	28	24.0	0.8	11.3		Rairat et al. (2020)
	2–4–8–15‰	SO‐FA	15	500–700	28	21.6–21.8–24.4–22.7	0.9–0.9–0.9–1.0	10.7–10.6–10.2–8.9		
Walleye	0‰	MO	10 × 10d	49–52	20–25	9.2–15.1			232–302	Kosoff et al. (2009)
Blunt‐snout bream	0‰	SO‐FA	25	50	18–28	5.9–6.2	6.0–2.8	26.8–16.1		Huang et al. (2019)
		MO‐FA	25 × 3d		28				198	
Crucian carp	0‰	SO‐FA	40	260	25	29.3	1.6	2.2		Zhao et al. (2011)
			10	260	10–25	2.4–3.5	2.8–3.7	22.9–9.6		Yang et al. (2019)
		MO‐FA	10 × 5d	330	10–25	4.0–1.7	2	19.9–12.9	72	Yang et al. (2020)
Catfish	0‰	MO	10 × 12d	916	18–20				96	Wrzesinski et al. (2006)
Korean catfish	0‰	SO	20	280	24	9.6	8			Park et al. (2006)
Rice field eel	0‰	SO‐FA	20	71	25	8.4	5	13.5		Xie et al. (2013)
Koi carp	0‰	SO	50	144	23–25	12.3	3			Yanong et al. (2005)
Three spot Gourami	0‰	SO	50	19	23–25	2.6	5			Yanong et al. (2005)
Rainbow trout	0‰	MO	10 × 10d	220	13				108	Di Salvo et al. (2013)
		SO	10	140	14–15	6.1	9			Pourmolaie et al. (2015)
		MO	15–20 × 10d	126–617	13				360	Meinertz et al. (2014)
Channel catfish	0‰	SO‐FA	10		25	7.6	9	9.1		Gaunt et al. (2012)
		MO‐FA	10 × 10d	612	25	9.7	8			Gaunt et al. (2013)
Atlantic salmon	30‰	SO‐FA	10	194	11	4	10.3			Martinsen et al. (1993)
	35‰	SO‐FA MO	10 10 × 10d	460 497	10	9.1 8.56	6			Horsberg et al. (1996)
Turbot	35‰	SO‐FA	100	101	16	55.4	12			de Ocenda et al. (2017)
Olive flounder	33‰	MO	20 × 3d	380	19	12.2	4		189	Lim et al. (2010)
Spotted halibut	31‰	SO‐FA	30	615	15–20	9.1–12.2	12	14.3–9.7		Chang et al. (2019)
Lumpfish	34‰	SO‐FA	10	114	12	3.6	21.2	21.2		Kverme et al. (2019)
Hybrid striped bass	26–30‰	MO	10 × 10d	384–426	20–25	1.8–3.5			17–62	Kosoff et al. (2009)
Gilthead seabream	28‰	MO	10 × 10d	150	27				95	Di Salvo et al. (2013)
Orange‐spotted grouper	33‰	SO‐FA	5–10	103–301	29	6–13	6	8.5–9.3		Feng et al. (2018)
	33‰		24	125	23	28	4	11.6		Feng et al. (2016)

Abbreviations: *C*_max_, maximum plasma concentration; FA, forced administration (oral gavage, syringe, hose); MO, multiple oral; SO, single oral; *t*_1/2b_, elimination half‐life of the drug; *T*_max_, time to reach *C*_max_; WT, withdrawal times.

Salinity has also been seen to affect the absorption and excretion rate of FLO, inducing faster elimination in seawater compared to fish kept in freshwater. Indeed, Rairat et al. (2020) reported higher C_max_ plasma values and faster elimination rates in Nile tilapia held at higher water salinity (C_max_: 21.6 μg/ml vs. 22.7 μg/ml and *t*_1/2b_: 8.9 hr vs. 10.7 hr at 2‰ and 15‰, respectively). The authors suggested that salinity‐dependent drug elimination may be also related to salt excretion in seawater fish. Differences in the excretion pathway of FLO between fresh and marine environment, as related to gill versus bile excretion, have been stated by Feng et al. (2008).

The large variations in pharmacokinetic parameters found in the literature may be also the result, among other biotic and abiotic factors, of the different drug administration routes and incorporation methods used to prepare the medicated diets. For example, Horsberg et al. (1996) observed higher C_max_ values achieved in a shorter time compared to the corresponding values measured in the study of Martinsen et al. (1993), when the same dose of FLO was offered by oral gavage in Atlantic salmon in oil‐coated and mixed medicated diets, respectively. Even though the experimental set‐up of the aforementioned studies was not identical, these observations partially justified the above assumptions. The peaked FLO levels were observed as early as 4 hr in European seabass which is in agreement with the majority of the pharmacokinetic studies stated (1.6 –21.2 hr) in Table 4. The measured *t*_1/2b_ of FLO in European seabass (13.0 hr) indicates a relatively fast elimination profile and is within the range of values found in other farmed fish (8.5–39 hr) (Table 4), however, this estimation should be treated with caution due to the limited range of examined time points. It is noteworthy that the residual European seabass plasma concentrations of thiamphenicol, an almost chemically identical amphenicol, were in accordance with the FLO peaked levels measured in the current study, 24 hr post treatment (Intorre et al., 2002). Specifically, the thiamphenicol levels in European seabass plasma 24 hr post treatment ranged from 0.3 to 1.1 μg/ml and from 0.4 to 1.2 μg/ml, after a multiple dosing regimen of 15 and 30 mg/kg for five consecutive days, respectively, reflecting similar depletion properties among the two amphenicols in fish circulation.

The WTs of the parent FLO and its major metabolite were also calculated in European seabass herein. Admittedly, the latter is of great interest as a marker residue for FLO even though it has been reported that it virtually lacks antibacterial activity (Park et al., 2006). The Committee for Veterinary Medicinal Products (CVMP) has identified the marker residue of FLO in fish as the sum of the parent compound and its metabolites in the target tissue (muscle plus adherent skin in natural proportions) (EMEA, 2002). The depletion trial herein indicates that the elimination of FLO and its amine from the edible tissues of European seabass is rapid, with measured WTs of approximately two days (or 46.3 dd) against an MRL of 1,000 μg/kg (EMEA, 2002). Although there were only few measurements above MRL, the mean concentrations were below the threshold in all selected sampling points. Ideally, measurements of at least two time points should have been above MRL (U.S. FDA, 2018), therefore our WT estimation should be considered as preliminary. Comparably, the WTs of FLO in gilthead sea bream muscle plus skin dropped below MRL on day two post treatment after a dosage of 10 mg/kg fish for 10 days at 27°C (Di Salvo et al., 2013). Generally, the differences in WTs of FLO among several studies (Table 4) are, in addition to variation in experimental set‐up, also due to species‐specific differences. Moreover environmental parameters such as water temperature and salinity have also proved to have an effect on the drug excretion pathway. In particular, Feng et al. (2008) reported that the primary route of excretion of FLO and its metabolites following a single oral dose of 10 mg/kg fish in freshwater hybrid tilapia is the bile duct, whereas in seawater tilapia, is mostly the gills. Additionally, Lim et al. (2010) demonstrated higher tissue concentrations of FLO in gills than in the other tested tissue samples in olive flounder *Paralichthys olivaceus* following multiple oral administrations at 20 mg kg^−1^ day^−1^ for three consecutive days. These findings indicate that the elimination of the drug would probably be faster in seawater species compared to the freshwater species. Indeed, the literature has revealed that the WTs of FLO were shorter in marine farmed fish (16–189 hr) compared to those calculated in fresh water (72–360 hr) (Table 4), which is consistent with the results of the present study.

In conclusion, FLO was readily absorbed in circulation and rapidly eliminated from European seabass edible tissues. Minimum inhibitory tests against important bacterial pathogens of European seabass and more importantly, specific clinical trials are needed to acquire a thorough profile of the antibacterial value of FLO. A double FLO dosage, administered twice a day may lead to enhanced circulatory drug levels in European seabass, but this has yet to be experimentally verified.

## AUTHOR CONTRIBUTION

**Dimitra Kogiannou:** Conceptualization; Formal analysis; Investigation; Methodology; Supervision; Validation; Writing‐original draft; Writing‐review & editing. **Chrysanthi Nikoloudaki:** Investigation; Resources. **Pantelis Katharios:** Investigation; Methodology; Writing‐original draft; Writing‐review & editing. **Adriana Triga:** Formal analysis; Investigation. **George Rigos:** Conceptualization; Supervision; Writing‐original draft; Writing‐review & editing.

### Peer Review

The peer review history for this article is available at https://publons.com/publon/10.1002/vms3.415.
